# How can post-COVID care be improved using patient experiences with received care and perceived health? A qualitative study of focus groups with 30 patients having post-COVID in the Netherlands

**DOI:** 10.1136/bmjopen-2024-090771

**Published:** 2025-09-21

**Authors:** Jeroen Gruiskens, Annerika HM Gidding-Slok, Thijs van Meulenbroek, Ivan Huijnen, Jean W M Muris, Jeanine Verbunt, Onno CP van Schayck, Jako S Burgers

**Affiliations:** 1 Department of Family Medicine, Maastricht University, Maastricht, The Netherlands; 2 CAPHRI School for Public Health and Primary care, Department of Family Medicine, Maastricht University, Maastricht, The Netherlands; 3 Department of Rehabilitation Medicine, Maastricht University, Maastricht, The Netherlands; 4 HAG, Maastricht University, Maastricht, Netherlands

**Keywords:** Post-Acute COVID-19 Syndrome, Chronic Disease, COVID-19, Organisation of health services

## Abstract

**Abstract:**

**Objectives:**

To provide bottom-up guidance on improving post-COVID care using patients’ experiences with received care and their perceived health

**Design:**

Qualitative study design using focus group interviews

**Participants:**

30 patients with post-COVID condition recruited through purposive sampling based on patient complexity and diversity

**Results:**

Three dimensions for potential improvements of post-COVID care were identified: (1) building, supporting and maintaining patient resilience, (2) redesigning healthcare pathways to meet patient needs and (3) embedding post-COVID care in health systems and organisations. A conceptual framework that could guide improvements in post-COVID care was developed.

**Conclusion:**

This study revealed several opportunities for improving and implementing post-COVID care following a person-centred approach in multidisciplinary integrated care pathways with an integrative vision of health.

**STRENGTHS AND LIMITATIONS OF THIS STUDY**

- Rigorous analytical methodology was used: Gioia’s approach and Krueger’ framework.
- The diverse population sample included participants from all levels of patient complexity.
- Focus groups allowed for constructivist development of usable concepts and integration with expert knowledge and scientific literature.
- Data collection took place in late 2022, and post-COVID care has been developing since then; thus, data might not be representative anymore for the current state of post-COVID care.

## Introduction

 The 2020 global pandemic caused by the SARS-CoV-2 virus included 776 million confirmed cases of COVID-19 and over seven million deaths.^1^ In Spring 2020, reports describing patients with persistent symptoms after an initial infection started to emerge.^2^ Not long after, the WHO introduced classification codes for the post-COVID condition,^3^ defined as *“the continuation or development of new symptoms 3 months after the initial SARS-COV-2 infection, with these symptoms lasting for at least 2 months with no other explanation”*.^4^ Common symptoms of post-COVID syndrome include fatigue, cognitive problems and pain, as well as many other symptoms.^5 6^ Currently, over 200 symptoms have been associated with post-COVID, leading to pervasive disability affecting everyday functioning.^7 8^ The prevalence of post-COVID varies from 45% to 50% of affected patients.^5 6 9^

Despite vast scientific research, the pathology of the disease is not completely understood, and research is complicated by the lack of consensus on the definition of the disease and plurality of symptoms.^710^,^14^ Moreover, recent systematic reviews found only weak to moderate evidence for effective treatment.^15^,^17^

Although the management of post-COVID syndrome is complex, effective care pathways should be developed as the syndrome is associated with a huge disease burden, high healthcare costs and loss of productivity.^18^

Using patient experience to co-create and optimise care pathways is an effective way to improve quality of care, patient satisfaction and care coordination, as it helps in centralising care around patients, as the Dutch Integral Health Accord implies.^19^,^22^ Therefore, a qualitative study was conducted with patients experiencing post-COVID, focusing on their experiences with their condition, related health needs and received care. We aimed to develop a guide that can be used in co-creating improved post-COVID care pathways. This research complements other scientific endeavours conducted in a larger research project that will evaluate the effectiveness of improved post-COVID care pathways embedded in a regional care network.

## Methods

### Study design

A phenomenological design was employed in exploring patient experiences, within a constructivist paradigm,^23^ to allow for the construction of knowledge arising from the dialogue between study participants. Semi-structured focus groups were chosen to allow for elucidating shared consensus and opinions, as the patient population is greatly heterogeneous in its presentation. Furthermore, focus groups allow for shared construction and co-creation of ideas and concepts through the heuristic interaction between different individuals.^24^ To ensure a controlled level of heterogeneity,^24^ four focus groups were organised, with participants having different degrees of disability complexity/related to disease complaints, as presented in table 1.

The study was approved by the local ethical review board (METC- 2022–3439). The study was funded by ZonMw grant number 50-56300-98-6061. Reporting was guided by the Consolidated criteria for Reporting Qualitative research checklist,^25^ given in online supplemental material 1. A topic guide and focus group protocol were developed (online supplemental material 2).

**Table 1:** Levels of patient complexity

Levels of complexity	Associated care pathway
1	Treatment in general practice
2	Monodisciplinary treatment by an allied healthcare professional
3	Multidisciplinary treatment by an allied healthcare professionals
4	Treatment in an outpatient rehabilitation centre

Levels of patient complexity. Patients are allocated to one out of four care pathways based on their level of complexity, which is associated with the scope and intensity of care provided in the respective care pathway.

### Sampling

To recruit participants, an online quantitative survey was conducted using an adapted version of the Assessment of Burden of Chronic Conditions-COVID (ABCoV) tool.^26^ The ABCoV tool was developed in 2020 to evaluate the burden of patients with post-COVID, using patient-reported outcome measures (PROMs) across 29 statements, and has since been used in studies as a measurement tool to assess COVID-19-related patient burden.^26^ We updated the original tool by adding additional questions according to findings from a literature review and data extracted from the previous use of the tool and developed an algorithm to determine patient complexity. In August 2022, we posted an open invitation for study participation on the website of the Dutch Lung Fund, a healthcare fund and patient organisation for patients with pulmonary diseases. A brief explanation of our research and our aims was provided. In total, 247 patients with post-COVID condition responded and were asked to complete the online questionnaire. An informed consent and patient information folder was distributed, detailing the outline and goal of the study. In total, 103 patients completed the questionnaire. Then, purposive sampling was conducted to recruit a balanced and diverse mix of patients for each level of complexity with regard to age, sex (male, female or otherwise) and educational status. Participants were allocated to one of the four focus groups. Based on the data retrieved from the open applications per mail, in which some patients extensively wrote about their problems, data retrieved from the ABCoV tool and a stakeholder meeting of a larger research project in which patients participated, a final version of a topic guide was verified by all authors involved. At the time of recruitment and data collection, no patients were using digital post-COVID care.

### Patient and public involvement statement

Participants were not directly involved in the study design; however, patients were given the option to choose between one-on-one interviews or participation in a focus group. Patients were recruited through a multi-tier sample process and drawn from a sample population of patients involved in a Dutch Post-COVID patient platform, ensuring representation of the general post-COVID patient population. This study used the data retrieved from the open applications per mail, in which some patients extensively wrote about their problems, data retrieved from the digital Assessment of Burden of Chronic Conditions Post-COVID filled in by the patients and a stakeholder meeting of a larger research project in which patients participated to help guide the formulation of a topic guide. We intend to disseminate the final paper and results to the national Post-COVID NL patient platform and the participants. Currently, our results are also used in the development of a national post-COVID care network.

### Data collection

Focus groups were organised through a secured online video connection to minimise the burden of participation. All focus group interviews were recorded with participant permission. Data collection took place in November and December 2022. Video recordings were transcribed verbatim. The focus group meetings with a total of 30 participants took between 1.5 and 2 hours, with breaks after 30 min. A moderator (JG) led the group discussion, supported by a scribe (AGS or TM). JG is educated and trained as a health scientist and healthcare consultant with previous experience in conducting interviews and as a researcher. AGS is a researcher in health science with experience in conducting qualitative research. TM is a researcher and physiotherapist and trained in conducting interviews. The sex of the researchers was not considered important. The researchers had no prior relationship with the participants. Patients had been supplied with a patient information brochure, which explains the research, its aims and the data collection method. Before the session was recorded, the moderator explained the research again, noting that the session would be recorded, and asked whether patients felt comfortable with it. The moderator also explained that the participation was entirely voluntary, and patients could leave at any time they wished. Two participants dropped out during the interviews because they felt emotionally overwhelmed and overloaded. These patients were asked during the sessions whether they had any support available to them, and the moderator contacted these patients after the session to check up their well-being. The data from these participants were included in the analysis, as they would significantly help us understand the pervasive nature of the post-COVID condition and its psychosocial burden. During the sessions, the moderator, assisted by the scribe, led the group discussion by asking questions and offering prompts. Patients were allowed to semi-guide the group discussions and add new topics during the focus group meetings. At the end of each focus group meeting, we reflected on what was said, and patients were given a chance to provide further feedback and express directly their opinion on how to improve care, which answers were taken into consideration for future focus groups and as extra data in the analysis. A member check was sent out to all participants in the focus groups; no changes had to be made according to feedback received. No repeat interviews were conducted.

### Data analysis

Data analysis was conducted using Atlas.Ti 22.1. Kreuger’s framework analysis and Gioia’s approach were used to analyse the data.^27 28^ These approaches ensure rigour and reproducibility of the results. The transcriptions were coded using Gioia’s approach, which allows for an abductive approach to thematic analysis and takes a disciplinary stance during the analysis. The approach allows for integrating participant data with scientific literature and expert knowledge. Going back and forth between all data collected, codes and categories were iteratively grouped into emerging themes and aggregated into dimensions. The first author produced a data structure based on the categories, emerging themes and aggregate dimensions. Compelling or representative quotes were extracted from the transcript. The data structure and quotes were integrated into a data table. The initial results and data table were presented to a second analyst, who independently went through the transcripts and compared the initial results with her perspective. The first and second authors then discussed the data table and associated quotes and worked towards consensus. When consensus was reached on the representativeness and robustness of the results, the data structure and table were adapted.

## Results

**Figure 1:**
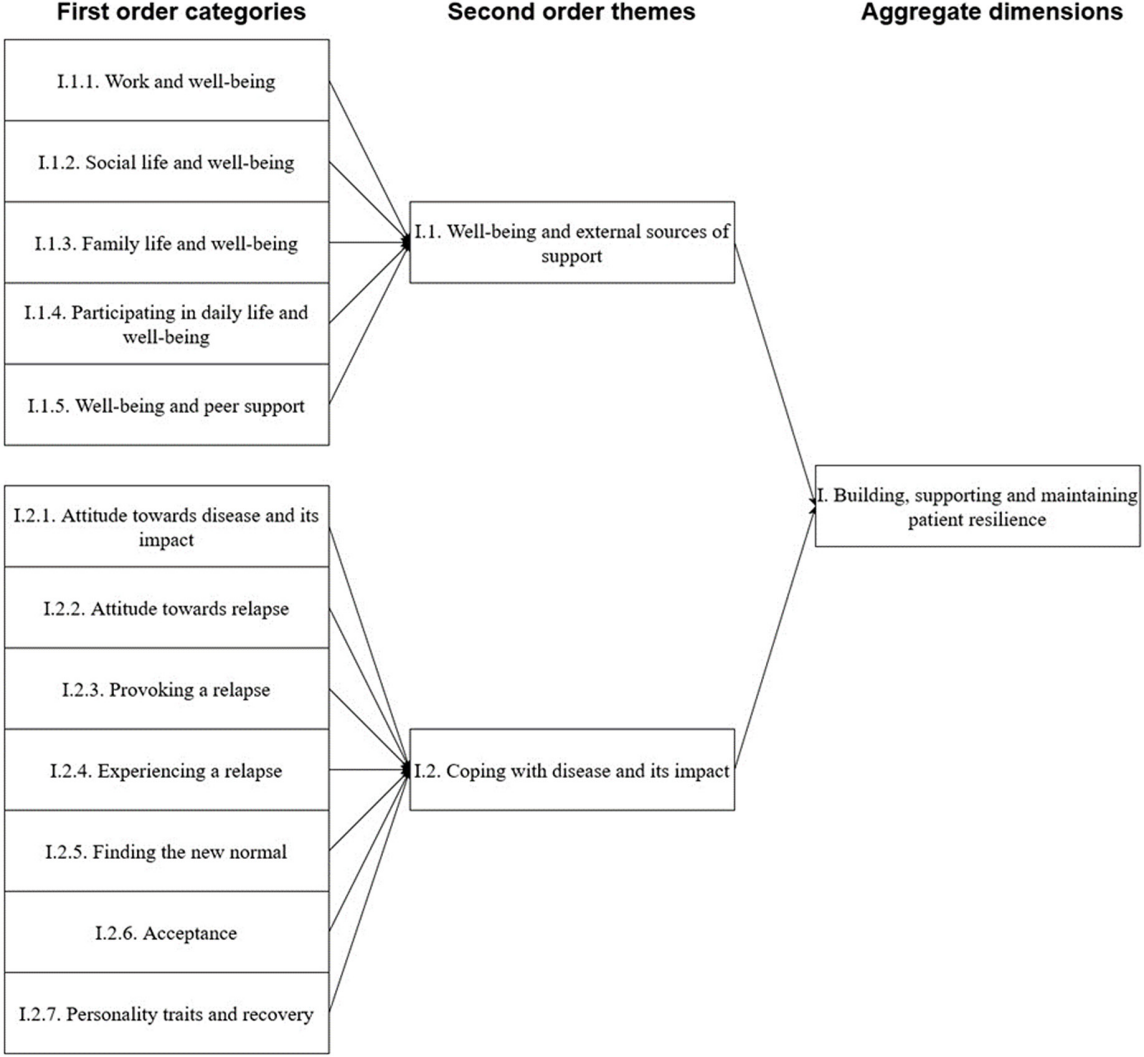
Data structure of the first aggregate dimension. This figure shows the building blocks of the data tree that suggest a role for building, supporting and maintaining patient resilience.

After obtaining informed consent, 30 patients were allocated to focus groups, divided into four focus groups of 5–11 participants. Across and within-group analyses did not yield different results. Basic characteristics of the participants are presented in table 2. Figures1^3^ provide a graphical overview of the data structure, which is supported by the data table in online supplemental material 3.

**Table 2:** Basic patient characteristics (n=28*)

	N (%)	Mean (SD)
Sex		
Male	6	
Female	24	
Age (years)		47.4 (10.2)
<45	9 (30%)	
>45	21 (70%)	
Educational level		
Secondary vocational education	11 (36.7%)	
Higher professional education	8 (26.7%)	
Postgraduate continuing professional education	5 (16.7%)	
University	6 (20.0%)	
Previous admission to hospital for acute COVID-19	3	
Experience of limitations in daily life†		3.1 (1.5)
Never/rarely	5 (17.8%)	
Sometimes	2 (7.2%)	
Regularly	5 (17.9%)	
Often	9 (32.2%)	
Most of the time	6 (21.4%)	
Always	1 (3.6%)	
Post-exertional malaise		
Never	2 (6.7%)	
2–3 hours	7 (23.3%)	
4–9 hours	6 (20.0%)	
10–15 hours	1 (3.3%)	
15–23 hours	3 (10.0%)	
≥24 hours	9 (30.0%)	
Frequency of experiencing tiredness		4.2 (1.9)
Never	2 (7.1%)	
Rarely	1 (3.6%)	
Sometimes	3 (10.7%)	
Regularly	2 (7.1%)	
Often	6 (21.4%)	
Most of the time	4 (14.3%)	
Always	10 (35.7%)	
Time past since start treatment (months)		19.8 (9.9)

Information about patient characteristics was gathered using a modified version of the ABCoV tool.

* 28 patients provided data via an online questionnaire and were invited to participate based on their complexity; two patients were recruited through snowball sampling.

† Average experienced limitations performing heavy exercise, moderate exercise, daily activities and engaging in social activities.

**Figure 2:**
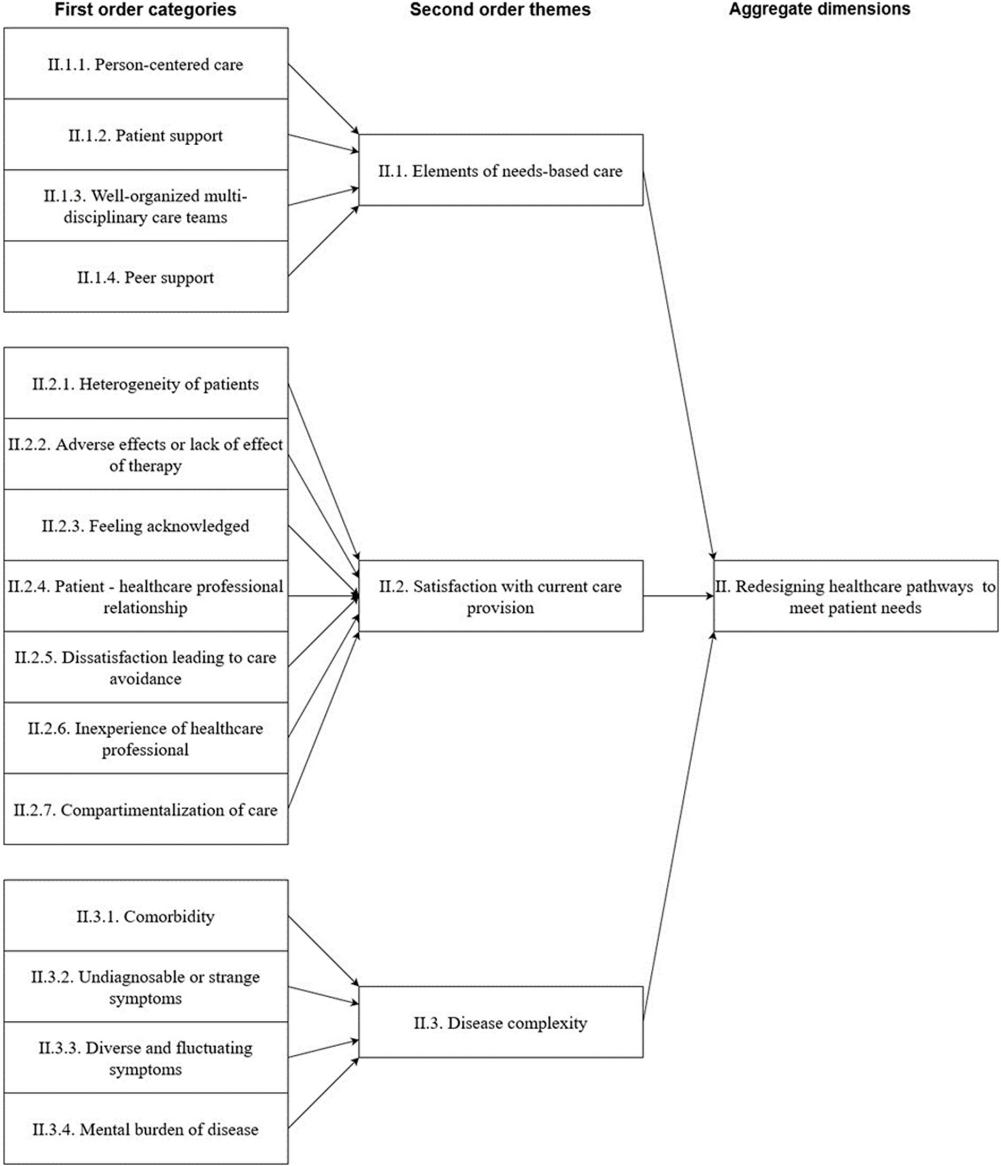
Data structure of the second aggregate dimension. This figure shows the building blocks of the data tree that suggests redesigning healthcare pathways to meet patient needs.

**Figure 3:**
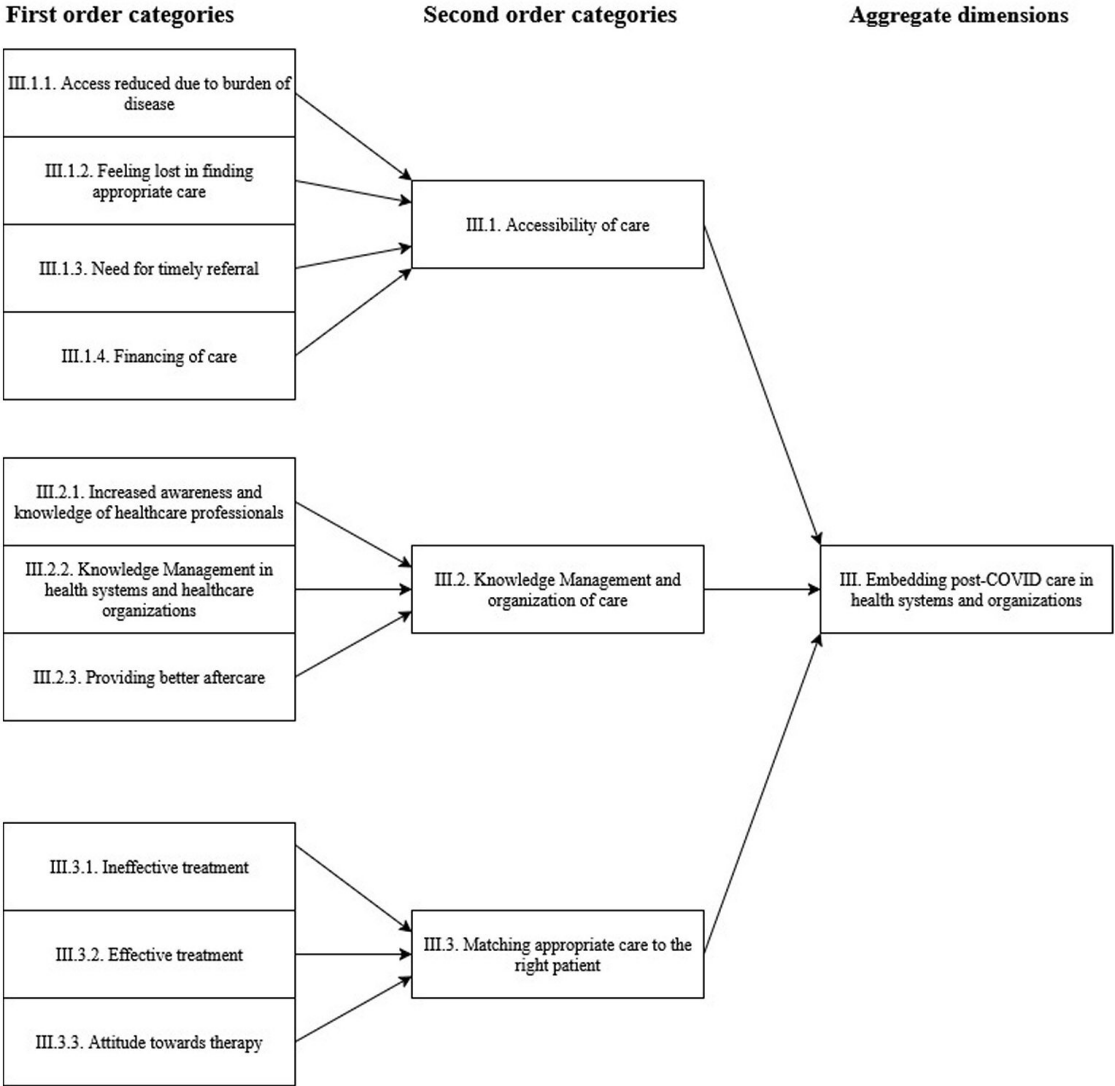
Data structure of the third aggregate dimension. This figure shows the building blocks of the data tree that suggest how post-COVID could be embedded in health systems and organisations.

Three dimensions related to post-COVID care that could be improved were identified based on our analysis of the experiences of patients with post-COVID. The results presented here were selected based on the data saturation reached; data saturation was defined as themes identified as consistent within focus groups or present across focus groups.^29^ Our main findings were described first, and our findings were integrated into a framework that might guide the development of post-COVID care pathways.

### Building, supporting and maintaining patient resilience

In our analysis, patient resilience might be a core determinant of recovery. When participants lacked the support of external sources or showed signs of maladaptive or non-constructive coping strategies, participants seemed to struggle more with health and recovery. Developing effective coping strategies or receiving support from their direct environment appeared to be beneficial for the well-being of participants.

### Well-being and external sources of support

The well-being of participants appeared to be related to the presence of external sources of support, such as work, social life and family life. These sources appeared either to provide support, for example, when family members were supportive of the participant in their recovery, or a source of stress, for example, when participants were unable to function at their job. The well-being of patients appeared to be affected by the presence of external sources of support and whether these sources were beneficial to their recovery.

Participant 1, female: *(About coping with hard times) “Lucky for me, I have a very warm family and my husband and my children and a couple of really nice friends”.*

### Coping with disease and its effects

Participants had different attitudes towards their disease, related disability and relapse. Participants who mentioned their recovery was slow or even stagnant appeared to have developed coping strategies that either rationalised or justified behaviour that would lead to a relapse or maintained coping strategies from their past, which led to relapses. Relapses, such as perceiving increased symptoms and/or reduced functional status, were often associated with over-exertion or crossing one’s boundaries or limitations. In some cases, participants mentioned that they sought to go beyond their limitations purposively. Furthermore, participants found it difficult to accept their new reality; however, when they learnt to cope with their disease and their disabilities, they found some relief.

Participant 2, female: *“I fell a few times to be honest, and then I began to understand. The occupational therapist helped me notice how my body felt when I took a few steps back, compared with when I constantly pushed my limits. Since then, I have been able to manage it better”.*

### Redesigning healthcare pathways to meet patient needs

Participants agreed that although their condition had similarities between subjects, a lot of benefits could be gained when care was built around patient needs, implying a desire of patients to receive person-centred care. Such care, given in multidisciplinary teams, would better match their health needs. Such teams would be able to address the wide variety of symptoms requiring multiple care expertises. Moreover, such care pathways would avoid the negative care experiences that our participants talked about. In care pathways built around person-centred care, disease complexity would be considered, and participants would feel more acknowledged and heard by their healthcare professional.

### Elements of needs-based care

There was consensus that the disease varied for each individual, and no single solution would be beneficial for all cases. Participants seemed to find it important to receive person-centred care that considers the heterogeneity of the patient population. In many cases, participants had received care from a multitude of healthcare professionals. Participants expressed positive experiences with organisations that provided patient support and spoke about missing a case manager. Given the lack of a one-size-fits-all solution, participants agreed that care should be delivered by well-organised multidisciplinary teams that determine the best approach for guiding and treating each patient.

Participant 3, female: *“Speaking from my own experience, I would have loved someone with a multidisciplinary perspective, someone who would not just do one piece of the problem”.*

### Satisfaction with current care provision

Many lessons can be learnt from the experience of participants with the care they provided. For example, many participants had negative experiences with receiving physiotherapy that was mainly focused on training their bodies. They attributed the adverse effects of this therapy to the lack of knowledge of healthcare professionals, which seemed to frustrate participants. This frustration seemed to be compounded by the notion that care was fragmented; as a result, care satisfaction and quality were reduced. Participants sometimes felt not taken seriously by healthcare professionals or were alone in their situation, which negatively affected their recovery. In contrast, acknowledgement of their illness seemed very important for patients’ well-being and trust in their healthcare provider. Unfortunately, many participants did not progress in their recovery anymore and were reluctant to keep searching for help and therapy.

Participant 4, female: *“I won’t do physiotherapy again; it really destroyed me during the initial phases of my recovery.… My physiotherapist did a sort of e-learning course for post-COVID. I worked out twice a week for half a year with her guidance, and that went well, but after therapy, I was bed-ridden for the whole week.… After I stopped the physiotherapy, my health increased significantly”.*

### Disease complexity

Participants often suffered from comorbidities that began to appear after COVID-19, and they related many of their undiagnosable or strange symptoms to post-COVID syndrome. Many participants spoke about their healthcare professionals not being able to find a diagnosis that could explain their symptoms. In addition, participants reported their symptoms were diverse and fluctuated in time. They recognised many of the symptoms in the narratives of other participants, and all participants agreed that the disease burden had a very negative effect on their functional status, well-being and quality of life.

Participant 5, female: *(Patient lists a long list of symptoms) “It is all very strange and weird, and they (healthcare professionals) just don’t understand where it is coming from”.*

### Embedding post-COVID care in health systems and organisations

Although experiences with care effectiveness varied greatly, there were several barriers to receiving post-COVID care. The disability of the disease itself prevented participants from reaching specialised therapy centres. The absence of knowledge management and organisational management of care reduced the quality of care the participants received. According to participants, much could be gained by addressing these barriers, for instance, by gathering and centralising knowledge in expertise centres and improving the exchange of information and knowledge between individual healthcare professionals.

### Accessibility of care

Although participants were searching for a therapy that could help them in their recovery, in some cases, their illness prevented them from receiving therapy. Their condition hindered them, for example, in being mobile and fit enough to travel to a therapeutic centre. Frustrated with the lack of progress in their recovery and the lack of knowledge of their healthcare professionals, many participants mentioned that they had taken it on themselves to find appropriate therapy, for which some assertiveness was required. In a few cases, participants found a therapy that helped their recovery significantly, where previously their recovery had been stagnant or riddled with many relapses.

Participant 6, female: *“They (employer and insurance company) wanted me to enter a rehabilitation trajectory, but I had to go to a place outside my own hometown. Me, I can’t even drive properly yet!”*

### Knowledge management and organisation of care

When the participants were asked what they would like to change, they answered that they would like healthcare professionals to have more awareness and knowledge about the disease. By increasing the awareness and knowledge of healthcare professionals, adverse effects of therapy could be avoided, and patients would feel more understood. Such knowledge should be spread throughout organisations and among healthcare professionals. Care that would be optimised in its knowledge management and organised around collaboration would benefit from the accumulation of the field expertise of many healthcare professionals. Moreover, care should also be reimbursed properly, as in some cases, participants mentioned that therapy was inaccessible because of financial constraints. This varied also within care disciplines; speech therapy and physiotherapy were better reimbursed. Even when care had been provided, participants felt aftercare was lacking, and they felt abandoned.

Participant 7, female: *“One element is that nobody talks to each other (talking about communication between different healthcare professionals), so I brought the message to them myself, and I think I am still on top of my game, but I am still a lay person, and that’s difficult”*.

### Matching appropriate care to the right patient

Participants had varying treatment experiences. Physiotherapy was often mentioned as ineffective, and in some cases, the lack of effectiveness was attributed to the post-COVID condition itself. Participants seemed to hope that there would still be a curative treatment that could help most of them. In contrast with the physiotherapy experience, occupational therapy was often mentioned as very helpful. A few patients mentioned that after struggling for over a year, and even after going through rehabilitation in a medical centre, their condition did not improve until they had found a specific therapy themselves that quickly seemed to alleviate their symptoms, such as therapies designed for the treatment of post-traumatic stress disorder or mindfulness-based breathing exercises. Participants agreed with each other that treatment effectiveness is associated with very personal and individual health needs and with finding the right care provider.

Participant 8, male: “*I started to do certain exercises (breathing exercises). After 3 days, my dizziness was gone. After 2 weeks, I had clarity again, and after 3 months, I was 100% recovered”.*

### Framework for post-COVID care

**Figure 4:**
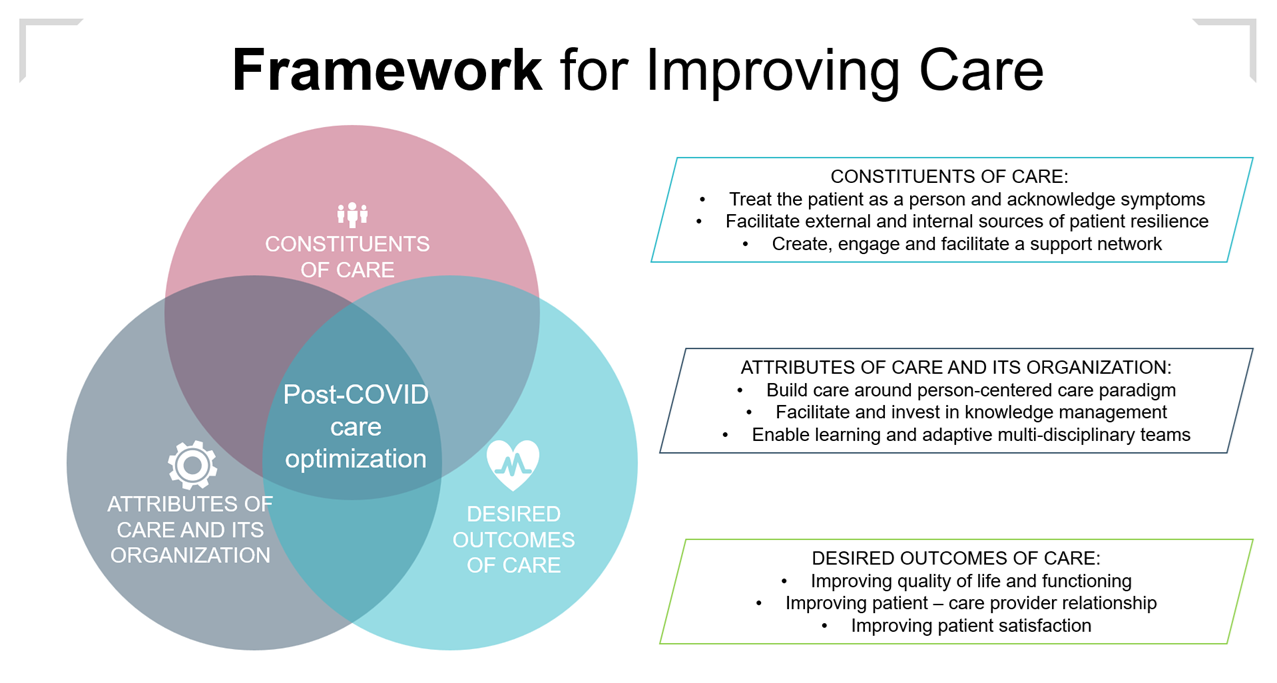
Proposed framework for improving post-COVID care. This figure shows the building blocks and their interrelations that might be used for improving post-COVID care.

The results of our analysis are interrelated and not limited by the data structure provided. Many categories developed in our analytical process correspond or relate to other emerging themes or aggregate dimensions than those under which they are initially grouped. To shape our results into a practical guide, a framework was developed based on the relations between categories, themes and dimensions. Although many frameworks exist for describing care quality improvements,^30^ our framework was developed using the conceptual basis of the framework described by Kates, Hutchison, O’Brien, Fraser, Wheeler and Chapman (2012).^31^ This framework was originally developed to describe elements of high-performing primary care. The COVID-19 pandemic and the subsequent post-COVID crisis have highlighted the importance of primary care in managing the burden placed on healthcare systems.^32^ This advocates a framework describing an approach that centralises care around high-performing primary care that is not limited to primary care organisations. In our framework, elements of care built around the three pillars were described: (1) constituents of care, (2) desired outcomes of care and (3) attributes of care and its organisation. Figure 4 illustrates the proposed framework.

## Discussion

This study led to the identification of three dimensions through which post-COVID care pathways might be improved. The first dimension describes the importance of patient resilience in their well-being and why care should support external and internal elements present in patients. This study suggests that bolstering both external (eg, social networks) and internal (coping mechanism) resources of resilience might improve patient well-being and functional status, leading to improved daily functioning. The second dimension describes why redesigning healthcare pathways to meet patient needs could improve care outcomes based on patient experiences with received care and their needs. This study indicates several opportunities to facilitate person-centred care, such as investing in patient–care provider relationships and taking into consideration and acknowledging the complexity of patient symptoms and care needs. The third dimension describes elements of care that should be given attention when implementing and embedding post-COVID care in current health systems and organisations, which include enhancing accessibility of care, optimising care organisation and knowledge management and facilitating appropriate care provision. The findings of this study seem to be in line with national developments within the Dutch knowledge platform post-COVID^33^ and the Dutch patient organisation C-Support’s Developmental Agenda COVID-19 recovery- and after-care.^34^

Our findings suggest an important role for person-centred care. Person-centred care is an important attribute of care value^22 31^ and is associated with a more positive perception of the quality of care and improved health outcomes.^30^,^3235^ As our analytical narrative and other studies with patients with post-COVID condition describe, post-COVID is a complex disease having a pervasive effect on all life areas and quality of life.^38^ Patients with post-COVID often have complex care needs,^39^ which require a person-centred, holistic approach.^40^ However, person-centred care can be hard to operationalize and implement in clinical practice.^41^,^44^

Using the 2001 definition of person-centred care proposed by the Institute of Medicine,^44^ “providing care that is respectful of and responsive to individual patient preferences, needs and values and ensuring that patient values guide all clinical decisions”, would prevent patients from feeling misunderstood or unheard, especially by their healthcare professionals,^45^ a frequently reported experience by our participants as well as by many other papers.^45^,^51^

Such care should specifically consider the importance of patient resilience. Despite the lack of a clear unified definition, studies show that patient resilience is an important predictor of health outcomes and quality of life in patients requiring long-term care.^52 53^ Resilience acts as a protective factor in the response to stressful life experiences and helps in the development of an adaptive response to a stressor, making use of both internal, such as coping strategies, and external resources, such as social support.^52 54 55^ Indeed, studies show that, for example, a lack of social support is associated with poorer well-being of patients with post-COVID.^56 57^ However, since a biomedical cause explaining the full range of symptoms of post-COVID has not yet been discovered, we provide this recommendation based on the current state of knowledge. Integrating new findings in future care remains vital. Care remains biopsychosocial, so it is important to remain vigilant about new developments and facilitate these in future care pathways. Studies have shown an association between maladaptive coping and progression of post-COVID symptoms such as fatigue,^58^ which has previously been reported in studies of factors affecting the recovery of other post-infectious diseases.^59^ Comparisons might be made with cases of patients suffering from rheumatoid arthritis developing kinesiophobia, resulting in poor physiological and psychosocial health and more perceived disability.^59 60^ A subset of patient may have personal barriers that increase their disability, such as the aforementioned kinesiophobia. This argues for care pathways that pay attention to how patients cope with their condition and, where appropriate, address patients’ coping mechanisms that hinder their recovery.

This study has similarities with studies among patients with chronic conditions, describing a link between health outcomes and patient resilience.^60^ Emerging research on post-COVID supports our argument that healthcare professionals should be aware of patient resilience, resources that contribute to patient resilience and support where needed. The desire to understand the biological underpinnings of post-COVID syndrome, and the related hope of many patients that a cure can be found, is very understandable and fuelled by the lack of medical understanding of and/or empathy of healthcare professionals for the disease causing uncertainty for patients.^60 61^ The dearth of research into the biomedical cause of post-COVID remains a mystery, causing further distress for patients. Phenomena such as the Q-fever outbreaks or chronic fatigue syndrome/myalgia encephalomyelitis show similarity with post-COVID in the search for a biomedical cause and the unmet needs of the affected patients.^62^,^66^

However, it might distract from evidence accumulated in the study of patients with complex and chronic conditions that developing resilience decreases treatment burden and improves well-being,^67^ and stress-coping strategies are a predictor of self-efficacy in patients requiring long-term care.^68^ In the worst case, such a cure may never be found, and a new reality must be accepted that the negative health outcome of post-COVID syndrome persists.

This study further indicated that in addition to the well-described opportunities to improve care,^46 66^ such as ensuring accessibility to care, person-centred care with an integral vision of health, care for patients with complex disability should be delivered by well-organised multidisciplinary care teams. These teams might benefit from knowledge management, as knowledge management can help organisations and teams in exchanging, disseminating and learning knowledge rapidly using the input of field experiences with care.^69^,^71^ Such practices would facilitate an open learning environment that quickly adapts to new insights gained from field experiences and thus would enable the bottom-up development of best practices. For instance, newly discovered biomedical findings could then be easily integrated into the existing knowledge base and protocols. Knowledge silos are a pervasive and persistent problem in healthcare,^72^ and this study shows that not only are patients aware of the existence and negative effect of knowledge silos on quality of care, but patients also wish such silos were removed. Digital assistive tools that support and help operationalise person-centred care and facilitate knowledge sharing between compartmentalised healthcare systems can help patients co-create their health and increase patient value and patient satisfaction by meeting patients’ needs, even if recovery is uncertain. Digital assistive tools, such as the ABCoV tool, could assist in providing person-centred care and facilitate challenging consultation topics such as addressing coping strategies.^73^,^75^ Currently, the use of digital post-COVID care is actively used in many health systems.^76^

### Strengths and limitations

Although focus groups might lack the opportunity for in-depth exploration of individual experiences, the focus groups were filled with rich data constructed through a mutual narrative shared between participants. Our data collection allowed for the collection of a vast amount of data. Furthermore, the interaction between participants strengthened the development of categories, themes and aggregate dimensions through confirmation or the adaptation of arising narratives during the group discussion. The vast amount of data collected in the four focus groups was congruent and across the group discussions, leading to satisfaction about data saturation.^25^ We furthermore used a topic guide developed by expert opinion and sent out member checks.

Our methods furthermore contribute to the development of usable concepts by allowing for an abductive reasoning, integrating participant data with academic literature and professional expertise, supported by a replicable analysis of the presented data. Gioia’s approach is particularly suited to increase robustness, credibility and rigour of qualitative research,^28^ and we believe that this approach helped us in avoiding common pitfalls of qualitative research such as the fragmentation of coding and lack of producing theoretical concepts.^77^

Despite achieving data saturation in our focus groups, the heterogeneity of patients and variations in levels of complexity, as well as the large total patient population, there could be a discussion about the theoretical sufficiency of this study. However, the common experiences of our participants suggest that our data led to findings relevant to all levels of patient complexity. Our findings could be complemented by the perspective of healthcare professionals and might be an interesting avenue for future research.

Although we were satisfied with the sample recruited, patients were sampled from an active post-COVID patient platform, which might lead to some bias, as patients involved in such a platform usually are more engaged. Although persistent post-COVID symptoms are more common in female patients,^78 79^ and our sample was predominantly female, valuable insights on sex differences in care experiences and outcomes might be missing from our research and could be a topic for future research.

Although the data we collected in late 2022 and the treatment protocols and care pathways have evolved since then, our recommendations in this paper appear to support current national trends in post-COVID response.

### Conclusion

This study shows potential opportunities for improving post-COVID care from a patient perspective. We propose developing these opportunities in three dimensions: (1) building, supporting and maintaining patient resilience, (2) redesigning care pathways to meet patients’ needs and (3) embedding post-COVID care in health systems and organisations. The framework synthesised from our findings highlights the importance of person-centred care delivered by learning and adaptive multidisciplinary care teams, supported by knowledge management.

## Supplementary material

10.1136/bmjopen-2024-090771 online supplemental file 1

10.1136/bmjopen-2024-090771 online supplemental file 2

10.1136/bmjopen-2024-090771 online supplemental file 3

## Data Availability

Data are available upon reasonable request.
