# Supplementary material for: How can post-COVID care be improved using patient experiences with received care and perceived health? A qualitative study of focus groups with 30 patients having post-COVID in the Netherlands
Source: BMJ Open. 2025 Sep 21;15(9):e090771. doi: 10.1136/bmjopen-2024-090771 (PMC12458768; doi:10.1136/bmjopen-2024-090771)
Supplement: online supplemental file 1 [file bmjopen-15-9-s001.docx]

**Supplementary Material 1**

**Consolidated criteria for reporting qualitative research studies (COREQ): 32-item checklist**

| **No** | **Item** | | **Guide questions/description** |
| --- | --- | --- | --- |
| **Domain 1: Research team and reflexivity** | | |  |
| *Personal characteristics* | | |  |
| 1 | | Interviewer/facilitator | Which author’s conducted the interview or focus group: see author contribution, page 1 |
| 2 | | Credentials | What were the researcher’s credentials: see page 1 |
| 3 | | Occupation | What was their occupation at the time of the study: see page 1 |
| 4 | | Gender | Was the researcher male or female: not relevant |
| 5 | | Experience and training | What experience or training did the researcher have?  1. JRJH Gruiskens: experience in qualitative research , working experience as healthcare consultant (change management, care pathway design, digital innovation, optimizing value-based healthcare, knowledge management, strategic management healthcare organizations, network building)  2. AHM Gidding-Slok: Public health scientist  3. T van Meulenbroek: Researcher and physiotherapist in a rehabilitation center |
| *Relationship with participants* | | |  |
| 6 | | Relationship established | Was a relationship established prior to study commencement? No, only contact for recruitment purposes |
| 7 | | Participant knowledge of the interviewer | What did the participants know about the researcher? E.g. personal goals, reasons for doing the research:  An informed consent was send out detailing the outline and purpose of the study |
| 8 | | Interviewer characteristics | What characteristics were reported about the interviewer/facilitator? E.g. Bias, assumptions, reasons and interests in the research topic: professional experience was reported, see page 6 |
| **Domain 2: study design** | | | |
| *Theoretical framework* | | | |
| 9 | | Methodological orientation and Theory | What methodological orientation was stated to underpin the study? E.g. grounded theory, discourse analysis, ethnography, phenomenology, content analysis: phenomenological orientation using constructivist paradigm (see page 5), abductive analysis by Gioia’s approach and Krueger’s Framework Analysis (see page 6) |
| *Participant selection* | |  |  |
| 10 | | Sampling | How were participants selected? E.g. purposive, convenience, consecutive, snowball: convenience sampling followed by purposive sampling (see page 5) |
| 11 | | Method of approach | How were participants approached? E.g. face-to-face, telephone, mail, email: open recruitment via online call, contact via mail |
| 12 | | Sample size | How many participants were in the study? 30 |
| 13 | | Non-participation | How many people refused to participate or dropped out? Reasons? None |
| *Setting* | |  |  |
| 14 | | Setting of data collection | Where was the data collected? E.g. home, clinic, workplace: online via secured videocall |
| 15 | | Presence of non-participants | Was anyone else present beside the participant and researchers? No |
| 16 | | Description of sample | What are the important characteristics of the sample? E.g. demographic data, date: See Table 2 |
| *Data* | |  |  |
| 17 | | Interview guide | Were questions, prompts, guides, provided by the authors? Was it pilot tested? Topic guide provided in supplementary material |
| 18 | | Repeat interviews | Were repeat interviews carried out? If yes, how many? No |
| 19 | | Audio/visual recording | Did the research use audio or visual recording to collect the data? Both audio and visual recording |
| 20 | | Field notes | Were field notes made during and/or after the interview or focus group? Yes |
| 21 | | Duration | What was the duration of the interviews or focus group? 1.5 to 2 hours |
| 22 | | Data saturation | Was data saturation discussed? Yes |
| 23 | | Transcripts returned | Were transcripts returned to participants for comment and/or correction: No |
| **Domain 3: analysis and findings** | | | |
| *Data analysis* | | |  |
| 24 | | Number of data coders | How many data coders coded the data?: one coder, one independent analyzer (see page 6) |
| 25 | | Description of the coding tree | Did authors provide a description of the coding tree? See Data Table |
| 26 | | Derivation of themes | Were themes identified in advance or derived from the data? Derived from the data |
| 27 | | Software | What software, if applicable, was used to manage the data? Atlast.Ti, word, Miro |
| 28 | | Participant checking | Did participants provide feedback on the findings? Member check was send out, feedback received |
| *Reporting* | |  |  |
| 29 | | Quotations | Were participant quotations presented to illustrate the themes/findings? Was each quotation identified? E.g. participant number: Yes, see main manuscript and data table |
| 30 | | Data and findings consistent | Was there consistency between the data presented and the findings? Yes |
| 31 | | Clarity of major themes | Were major themes clearly presented in the findings? Yes |
| 32 | | Clarity of minor themes | Is there a description of diverse cases or discussion of minor themes? Yes |
