# Supplementary material for: How can post-COVID care be improved using patient experiences with received care and perceived health? A qualitative study of focus groups with 30 patients having post-COVID in the Netherlands
Source: BMJ Open. 2025 Sep 21;15(9):e090771. doi: 10.1136/bmjopen-2024-090771 (PMC12458768; doi:10.1136/bmjopen-2024-090771)
Supplement: online supplemental file 2 [file bmjopen-15-9-s002.docx]

**Supplementary Material 2**

**Focus group topic guide PINCOR**

**Post-COVID patients**

| **Titel** | Focus group protocol PINCOR WP1 and WP3: patients |
| --- | --- |
| **Written by** | Jeroen Gruiskens, Maastricht University |
| **Version** | 1.0 |
| **Date** | 09-09-2022 |

| **Aims** |  |
| --- | --- |
| **1** | Map, explore and investigate symptoms post-COVID patients with which they present themselves to healthcare professionals |
| **2** | Existing data about spectrum of symptoms of post-COVID patients extracted from the database of the ABCoV-tool, (inter-)national guidelines and academic literature cross-reference with patient experiences, to the aim of further develop the Assessment of Burden of Chronic Conditions post-COVID tool and the referral index |
| **3** | To explore the effectivity, desirability and quality of received post-COVID care using patient experiences |
| **4** | To explore the care needs and wishes regarding future treatment of patients |

| **Method** |  |
| --- | --- |
| **1** | Qualitative interviews using focus groups |
| **2** | 4 focus groups with patients, diverse background, diverse in complexity |
| **3** | Duration 1,5 – 2 hours |
| **4** | Online video conference, recorded digitally |

| **Topic guide** |  |
| --- | --- |
| **Introduction** |  |
|  | 1. Word of welcome 2. Introduction Moderator and Scribe 3. Introduction round participants 4. Reaffirm agenda of the focus group |
| **Administrative and informed consent** |  |
|  | 1. Further explanation research 2. Approval participation, ask for verbal confirmation |
| **Rules and procedure** |  |
|  | 1. Explain procedure focus group |
|  | 1. Set ground rules and explain tasks moderator and scribe |
| **Execution focus group** |  |
|  | 1. State recording starts |
| **Guiding through research themes** |  |
|  | 1 What complaints are present in patients?  2 What limitations do patients experience?  3 How do complaints and limitations affect  patients' daily lives?  4 How do patients deal with their experienced complaints and limitations?  5 What experiences do patients have with the care they receive or have received?  6 What healthcare needs do patients have?  7 What wishes do patients have regarding their (future) treatment?  8 Which factors are appropriate for treatment?  9 What barriers do patients experience regarding their treatment? |
| **Example of questions** | 1 What complaints have you experienced after experiencing a COVID-19 infection?  2 Think back on the past year. What complaints did you have?  3 What are you most bothered by at the moment?  4 How does having Post-COVID affect your daily life?  5 In the past year, what have you experienced as the most stressful?  6 How do you deal with your complaints?  7 How do you respond when you suffer from complaints?  8 How do you view the limitations you experience?  9 How did you seek help for your complaints?  10 What care are you currently receiving for your complaints?  11 When do you use care for your complaints?  12 What would you like to receive care for?  13 What went well with the care you received?  14 What went wrong with the care you received?  15 uppose you could change healthcare for Post-COVID, and you could make one change in current healthcare. What would this change be? |
| **End of focus group** | 1. Final questions  - Reflecting on the focus group - Asking the amazement questions  1. Provide summary and ask for confirmation of participants 2. Final closing questions 3. Ending of focus group |
|  |  |
