# Supplementary material for: How can post-COVID care be improved using patient experiences with received care and perceived health? A qualitative study of focus groups with 30 patients having post-COVID in the Netherlands
Source: BMJ Open. 2025 Sep 21;15(9):e090771. doi: 10.1136/bmjopen-2024-090771 (PMC12458768; doi:10.1136/bmjopen-2024-090771)
Supplement: online supplemental file 3 [file bmjopen-15-9-s003.docx]

**Supplementary Material 3**

| **Table 2: Data table** | | | |
| --- | --- | --- | --- |
| **Aggregate dimension: I. Building, supporting, and maintaining patient resilience** | | | |
|  | ***Second-order theme: I.1. Well-being and external sources of support*** | | |
|  | | ***First order categories*** | ***Representative data*** |
|  | | *I.1.1. Work and well-being* | 1. “The fact that I don’t work anymore, that really still causes me a lot of grief. It remains very difficult...” [Participant starts to cry].   2. “I kept working very long after my corona infection,  still eight hours per day, but at a certain moment, I  kept working like that for four, five months, but at  one point, I just melted down. Then… [various  healthcare professionals] … told me I should really  start working less.”   1. “But also rebuild social life, because you really want to start working again … but with every little thing you do to much, you will relapse hard.” |
|  | | *I.1.2. Social life and well-being* | 1. “I can schedule my day pretty well, and that is how I endure, but with no social contacts during the week.” 2. “At one point, I withdrew from my circle of friends, because I had to decline [to invitations] and I noticed I had to skip all fun things, and I couldn’t participate. Even with how much understanding they had, I felt pressured, also because I put the pressure on myself, and I couldn’t really cope with that.” 3. “I need to make choices … Will I want to go to that event? Do I want to pay the costs of a relapse and be bedridden for two days?” |
|  | | *I.1.3.* Family life and well-being | 1. “I had great difficulty explaining it [their disease]… You simply don’t know what is going on yourself. … But these are also the people who help you through the ordeal.” 2. “My husband, he said just recently, that I was not the only one with the disease. He told me, we all have it. And for me it is sometimes unexplainable, so for them even more.” 3. [About coping with hard times] “Lucky for me, I have a very warm family and my husband and my children and a couple of really nice friends.” |
|  | | *I.1.4. Participating in daily life and well-being* | 1. “I have to embed and calculate such activities into my daily life… [talking about the costs of such activities] … But if I did such things, then… and I try to do such things, otherwise I don’t feel my life means anything anymore… but these positive things that make my happy, will cause a relapse.” 2. “I have a garden, and I used to be very active in it. It just won’t work anymore to do everything on a day what you were used to doing. Grocery shopping and going to stores, that is slowly getting better, even with all the stimuli, but I couldn’t do that any longer at one point. I was just broken.” 3. “And there are days, that I just do that, and then I will lay in bed for a couple of days, but those are conscious choices. A psychologist told me to do once per month something nice … and that I do, and I am really tired after it, but then I have experienced a little bit of happiness instead of staying in that bubble.” |
|  | | *I.I.5. Well-being and*  *peer support* | 1. “I agree with all the stories I heard here thus far. We could all shake each other’s hand … and anyway, it is nice to hear everybody has similar problems.” 2. “It is normal. I keep busy with Long-COVID patients. I see a lot of people. Then you think, thank god. I am not the only one.” 3. “And actually, fellow patients, that goes through the Facebook site, [having contact], but not that they are entire groups, although I would like that, to share experiences.” |
|  | ***Second-order theme: I.2. Coping with disease and its impact*** | | |
|  | | ***First order categories*** | ***Representative data*** |
|  | | *I.2.1. Attitude towards disease and its impact* | 1. “And [about learning to cope with their illness], that worked very well for me, it was not the path to complete recovery, but learning how to cope, I experienced it as very positive.” 2. “I became disabled to be honest. And I do take a positive look on life, like I am not going to hide in a corner, because nothing will come from that, because I learned during my rehabilitation to cope with my disability. But there is still a small piece in my head that wants so much more, and its hard to let that go sometimes.” 3. [About coping with disability] “I had help with that during my rehabilitation that I had this year, because I know it all, and I can name it all, but I just do not want to feel it, and I told that. I just don’t want it. I don’t want to be sick, and they actually showed – Actually they tell me too, but we can’t do anything anymore. What do you want? And then it clicked, like, they can’t make me better, also not at a doctor, but also not the people at the rehabilitation, so I have to.” |
|  | | *I.2.2.* *Attitude towards relapse* | 1. “But after that, I had, of course, a relapse. Very logical actually, and I don’t see it as something negative.” 2. “Those relapses really aren’t nice, and it is often very hard to accept that, but I think I am at a point I know it’s part of it.” 3. “At first, it was like, I kept thinking about when I did things and if I did those things, I would get a relapse. I was very strict on myself [due to mental health problems during adolescence] … but apparently, that was the mechanism that caused me to stay ill [when the fear of getting a relapse was let go and their disability was accepted].” |
|  | | *I.2.3. Provoking a relapse* | 1. “I can handle fatigue pretty well due to… [talking about experiences with an auto-immune disease the participant had before the COVID-19 infection] … well, actually, I cannot handle it pretty well, since I always cross my boundaries and then I collapse completely.” 2. “I consciously seek out my boundaries to – well doing fun things, otherwise my life would be worthless.” 3. “I schedule everything, and I keep watch on my boundaries, but I also try to break them and then I will just see what happens.” |
|  | | *I.2.4. Experiencing a relapse* | 1. “It felt like an elephant sat on my chest, and sometimes two at the same time. It is really not comfortable”. 2. [When talking about having a relapse] I will get inflammations again, I often have inflammation in my mouth, behind my jaw or behind my molars. And if I do too much, I get tinnitus, my fingers start to tingle, all kinds of strange things”. 3. “When you frequently experience relapses and you suffer the very grave consequences, it becomes traumatizing, and you will start to try to prevent it. As a result, you become hyper alert for overstimulation. And this kept me sick.” |
|  | | *I.2.5. Finding the new normal* | 1. “Now it goes ok in my new normal, but after 2,5 years I sometimes just don’t know what used to be normal. So I think I forget a lot of things, because I just don’t know how it was before and how it is to do sports, I don’t know anymore.” 2. “I don’t actually know what my old normal was, because I know it cognitively, but I can’t imagine it physically anymore.” 3. “And I was really struggling for my old self. So, I recognize [what other participant said], but I was so occupied with going back to what it once was.” |
|  | | *I.2.6. Acceptance* | 1. “It’s like he just said, it’s not accepting it, that’s like resigning, but it’s more learning to bear it.” 2. The fatigue, it took an eternity, and for me, it came down to a piece of acceptance. As soon as I accepted I could do less, everything became better.” 3. “But accepting it was very difficult, so I thought for a long time, no, I just want to do that and this, so I will just do it, even if I am tired. And then I had to recover for a week, but I couldn’t accept it. And when that acceptance came, and it took a long time for it to come, things gradually became better.” |
|  | | *I.2.7. Personality traits and recovery* | 1. “And I have a bit ADHD, because I used to be super active and very sportive, and my condition was very good.” 2. But I am a born and raised [Name of city], and that means no talking but cleaning. My grandmother was a very strong woman. If there is something wrong with you, don’t complain. Just go for it. And that means not listening to your body, because that’s something you needed to learn, and I should have told your employer, sorry, I just can’t do it any longer.” 3. [About what a therapist told a participant] “She told me she saw a lot of women, and they seem to have one common denominator, and that’s what I hear here also, people how naturally raise the bar very high for themselves, and demand a lot of themselves, don’t speak, but clean. It seems that is a common characteristic of all these people.” |
| **Aggregate dimension: II. Redesigning healthcare pathways to meet patient needs** | | | |
|  | ***Second-order theme: II.1. Elements of needs-based care*** | | |
|  | | ***First order categories*** | ***Representative data*** |
|  | | *II.1.1. Person-centered care* | 1. “It’s so important to look to what somebody is capable of, and what has priority and what will be the goal of our therapy … So I think really look at, ok, what matches with somebody?”. 2. [About suggestions for improving care] “And also using some kind of scale, that when you have like serious symptoms, you’ll for example go to a pulmonologist. But if you think, hey, my symptoms are not that strong, then you’ll have this option and that option. For example, the GP will talk with the patient about a roadmap, and the patient can co-decide … but I would like to go to a physiotherapist or occupational therapist for example.” |
|  | | *II.1.2. Patient support* | 1. “So, what do I miss? I think you actually miss someone that guides you in that process [finding appropriate treatment], and certainly in the beginning, because my apologies for my language, everything really sucks. You don’t have the energy to seek out information or to read, or to listen to something.” 2. “The first person who said to me: we are going to help you, you are not in this alone, that was such a relief”. |
|  | | *II.1.3. Well organized multi-disciplinary care teams* | 1. “What you mentioned, I think it is brilliant. That is what I would have wanted when I became sick. So, use a case-manager … that someone supervises all those different healthcare professionals in the care you received”. 2. “Speaking from my own experience, I would have loved someone with a multidisciplinary perspective, someone who would not just do one piece of the problem.” 3. [About a rehabilitation trajectory] “And I really liked that. You’ll have an occupational therapist, a physiotherapist and a psychologist that continuously treat you. |
|  | | *II.1.4. Peer contact* | 1. “It is normal. I keep busy with Long-COVID patients. I see a lot of people. Then you think, thank God. I am not the only one.” 2. “And actually, fellow patients, that goes through the Facebook site, [having contact], but not that they are entire groups, although I would like that, to share experiences.” |
|  | ***Second-order theme: II.2. Satisfaction with current care provision*** | | |
|  | | ***First-order category*** | ***Representative data*** |
|  | | *II.2.1. Heterogeneity of patients* | 1. “There is no readily available solution for everyone, everyone is unique, so you keep pioneering and seeking out that therapy that will help you in your recovery.” 2. “You don’t want to know how many people, how many colleagues of mine all have Long-COVID, but the one has it less as the other, their symptoms are all so different.” |
|  | | *II.2.2. Adverse effects or lack of effect of therapy* | 1. “I won’t do physiotherapy again, it really destroyed me during the initial phases of my recovery. … My physiotherapist did a sort of e-learning course for post-COVID. I worked out twice a week for half a year with her guidance, and that went well, but after therapy I was bed-ridden for the whole week. … After I stopped the physiotherapy, my health increased significantly.” 2. “Therapists still think about deconditioning and building up physical fitness. That is wrong … because you will literally be destroying yourself. … Despite that there are guidelines developed for physiotherapy in post-COVID Syndrome.” 3. “I spoke to my sports doctor. She previously gave me the advice that I needed to build up more intensely. And this week, I said, sorry I can’t do it anymore. And then … she offered her apologies and said she got new guidelines. And she said they shouldn’t be so strict to patients.” |
|  | | *II.2.3. Feeling acknowledged* | 1. “At one point, I came to a therapist and then suddenly it became known to me that many more people had similar problems and this disease. Thus, the part of getting recognition and acknowledgement, that meant a lot to me at [recovery organization].” 2. “The biggest limitation I had, was mostly the understanding of people. People really don’t understand. They have no idea. And the GP for example told me: you are a pioneer. I thought, I don’t want to be a pioneer. Please, can’t you find anything in medical manual? That was very hard for me.” 3. “I had the luck that my occupational physician understood me. They said, listen to your body. Talk to you supervisor about what you can and cannot handle and I’ll advise you to see a company social worker.” |
|  | | *II.2.4. Patient – healthcare professional relationship* | 1. “When he looked at me with a look like: “What are you doing here”, it gave me a very unsafe feeling and I started to question, like “there is something not entirely right with me?” 2. “I really have a very nice GP that worries a lot about me and tries to arrange all kinds of things. I went to see a pulmonologist, cardiologist, all kinds of people.” 3. “The occupational therapist was really great, especially not just the understanding, but also that everything was so recognizable.” |
|  | | *II.2.5. Dissatisfaction leading to care avoidance* | 1. “I don’t want to go each time to the general practitioner, because that costs energy as well, which I find a shame, because than I can do also other things.” 2. “And that’s what just was said. I can approach my GP, but they won’t find any new things, so I don’t know where to seek help anymore. … And that’s why I think: this is it then. Because it costs so much energy to keep going to a new medical specialist.” 3. “Who will monitor me? And all these complaints I have, they don’t get better, they are getting worse. Also, my hair. Then a GP says something like, if you get older, you might get that. Men have it too, getting bald. And then I think, just leave it. I often told my GP that I wouldn’t call anymore because it’s useless. And that’s poignant to me.” |
|  | | *II.2.6. Inexperience of healthcare professional* | 1. [On the question what they found lacking in current care provision] “Knowledge. My general practitioner literally asked me what I wanted from him. A referral? I would get it from him. He said: “You can get everything you want from me, because I don’t how I can help you”.   2. “I can ask for help at my pulmonologist. He acts. My  GP doesn’t. She doesn’t know. She asks me what she  should do.”  3. “I miss a place where people really know something  about these post-COVID symptoms.” |
|  | | *II.2.7. Compartmentalization of care* | 1. [About getting effective treatment from a psychosomatic therapist] “And that is what taught me the most, because she pierced through it, because I was someone who always put a lot of pressure on myself, and they did not see that at rehabilitation because a lot of different people looked at me and not one constantly seeing me.” 2. “I was thinking about what I noticed, but I of course don’t know if it is still like that, but when I came to see the GP, I did not know which paths you could take. That it would be also clear for a patient, like, these are the care pathways you might be able to take.” |
|  | ***Second-order theme: II.3. Disease complexity*** | | |
|  | | ***First-order category*** | ***Representative data*** |
|  | | *II.3.1. Comorbidity* | 1. “And what I got from the COVID-infection, is asthma. I didn’t have it before the COVID and now apparently this has developed.” 2. “Now I have shingles on my face, and each time, these little new things appear. You are so susceptible for everything, even the smallest things, your body will react to it.” 3. “I became lactose-intolerant. I also follow a special diet now. Because of COVID I gained almost 25 kilos in weight. I also am borderline diabetic, and I am working on that with the dietician and the physiotherapist with a FODMAP-diet.” |
|  | | *II.3.2. Undiagnosable or strange symptoms* | 1. “I have a lot of physical symptoms that are undiagnosable [Lists a number of symptoms], but they can’t find anything wrong.” 2. “[Patient lists a long list of symptoms] “It is all very strange and weird, and they [Healthcare professionals] just don’t understand where it is coming from.” 3. “And that fatigue, I always found that really hard, was not always measurable in me. My saturation was for example always normal. So, I was alone and I didn’t have any memory problems or other physical problems.” |
|  | | *II.3.3. Diverse and fluctuating symptoms* | 1. “My symptoms are very diverse.” 2. [Patient mentions a number of various symptoms] “Really a wide range of all kinds of different symptoms”. 3. “It changes … right after I got sick I had a lot of pain between my shoulders. That went away last year during summer, and this spring it is there again, and now it has faded away gain.” |
|  | | *II.3.4. Mental burden of disease* | 1. “I have difficulty with coping with it … Sometimes you just crack. Then you cry for a while and you have some good talks and then it fades a bit, but you just aren’t your old self anymore. And that is quite a mental burden to carry.” 2. “I have had help from a medical psychologist during the last three years. The pulmonologist had arranged him, and I could relieve my burdens there, but it is- If I pause to think about it, it is really hard.” 3. Especially walking stairs was really tough. I think walking the stairs was tough and the mental part became really hard at a certain moment, like, I am now still younger than thirty years old, I already see an occupational physician… How is this possible?” |
| **Aggregate dimension: III. Embedding post-COVID care in health systems and care organizations** | | | |
|  | ***Second-order theme: III.1. Accessibility of care*** | | |
|  | | ***First-order category*** | ***Representative data*** |
|  | | *III.1.1. Access reduced due to burden of disease* | 1. “They [employer and insurance company] wanted me to enter a rehabilitation trajectory, but I had to go to a place outside my own hometown. Me, I can’t even drive properly yet!” 2. [About going to rehabilitation] “The second time, I had to drive 45 minutes for that. Driving somebody just mentioned, I forgot to mention that that’s still very difficult. So that didn’t work out.” 3. “The physiotherapy wanted me to go back to rehabilitation, but I learned they did not want me there anymore because I didn’t progress anymore.” |
|  | | *III.1.2. Feeling lost in finding appropriate care* | 1. “Thus, you will start looking yourself. And that’s how I ended up at an occupational therapist and at a psychosomatic physiotherapist.” 2. “Most of all, I was extremely tired, so for a while I thought I might have had a burn-out. So, I talked about it with my occupational therapist, and she said, I don’t think so. And I didn’t think it either, but you just don’t know, so you will try everything.” 3. [About getting referrals] “I had to ask it every time myself!” |
|  | | *III.1.3. Need for timely referral* | 1. [About experiences with receiving care] “And not like it went with my GP: see how it goes and you will still have the same problems for another half a year.” 2. “My legs tended to collapse, and my GP actually attributed it to something like, you are weak. You have been so sick. Eventually, after almost a year, I got a referral to the neurologist, because I kept asking for it. And then they showed with an EMG that I have muscle weakness and neuropathy.” 3. “At a certain moment in time, I started to receive breathing therapy and actually- What you would like, is that a GP refers you faster when you have some problem and doesn’t tell you to wait and see what happens. My neuropathy, I had it for so long.” [without a diagnosis] |
|  | | *III.1.4. Financing of care* | 1. “And I had physiotherapy, but not the second time  because I did a self-test and that wasn’t accepted by  the insurance company. You really need an official  test if you want to follow a rehabilitation trajectory  again.”  2. “I went to see [a physiotherapist] him a couple of  times, and then it was like, contact professions  weren’t allowed, and she asks pretty steep prizes for  longer consultations. I thought, this is not helping.” |
|  | ***Second-order theme: III.2. Knowledge management and organization of care*** | | |
|  | | ***First-order category*** | ***Representative data*** |
|  | | *III.2.1. Increased awareness and knowledge of healthcare professionals* | 1. “What I would like to change, is knowledge … Just pure knowledge present at healthcare professionals and doctors about post-COVID … There are protocols and guidelines, and they don’t use them … A suspected post-COVID patient comes to them, and they just say: I don’t know.” 2. “I would like to change … that it is made clear that it is not just a mental problem. Even if you can’t find anything … you are still sick. But it’s not a mental problem. I would like general practitioners, occupational physicians, to get that it’s not a mental problem.” 3. “I just want to recover, and I also know, I first need to start taking care of myself, the thing you hear over here- There is not much being done. Doctors don’t know. So, my hope of recovering is vanishing, and I am really feeling bad about it, like, is this my life now?” |
|  | | *III.2.2. Knowledge management in health systems and healthcare organizations* | 1. “One element is that nobody talks to each other. So, I bring the message to them myself. And I think I am still on top of my game, but I am still a lay person, and that’s difficult”. 2. “I have to bring the news from the one to the other, indeed”. 3. “How useful would it be if you could collect all those experiences and all the knowledge from the whole country and put it in one care pathway?” |
|  | | *III.2.3. Providing better after care* | 1. [About rehabilitation trajectories] “And I am assertive of myself, so I search for it, when necessary, but now I feel like I am falling into a hole. My GP, if I call him or when I am there… they care for me, but I don’t have the feeling that there is somebody that concerns himself with my health … but there is no care provider who says: I have you, and I am there for you.” 2. “I would like it that care is something more long-lasting so they can follow you and not just for those three months”. 3. “For me, the rehabilitation stopped, because I had to stop … I felt abandoned … so for me, after-care can be better.” |
|  | ***Second-order theme: III.3. Matching appropriate care to the right patient*** | | |
|  | | ***First-order category*** | ***Representative data*** |
|  | | *III.3.1. Ineffective treatment* | 1. “What I noticed is that … I am not trainable. So repetitive physiotherapy did not lead to an increase in strength, which I am used to, because I always did sports.” 2. **“**In my opinion, there still isn’t anything comprehensive that could help us. I still have to hope that I recover a bit by something, but I still can’t find anything that help me progress.” |
|  | | *III.3.2. Effective treatment* | 1. I really had a lot of help from the occupational therapist. She is worth gold … She explained everything … and she helped me accept things.” 2. “I started to do certain exercises [breathing exercises]. After three days my dizziness was gone. After two weeks I had clarity again, and after three months I was 100% recovered.” 3. “The occupational therapy really helped me with my energy management because I had to make this energy planning, but they also work with this traffic light method, what really helped me like, ok, now you are in the orange zone. What helps you to go to green. And those are all things that you can think of yourself, but sometimes it is nice if somebody else then says, you need to do that now, and then you will be made aware. So that helped me. |
|  | | *III.3.3. Attitude towards therapy* | 1. [About following a second rehabilitation program]  “Occupational therapy, I had it a few times, but it was  more or less the same as what I already knew. So, I  quit that soon enough.”  2. “I stopped going to physiotherapy after a few times,  because that was just learning to build up really  slowly and that’s really hard for this ADHD person,  because I used to go from zero to one hundred.”  3. “I can’t complain about the care I received, only I  really regret that at a certain point the pulmonologist  again told me they couldn’t do anything for me  anymore. Then I think, is that true? And I still am not  convinced about that, but I just don’t know what I  should do to get rid of these disabilities, that you can  do stuff bending down. That just isn’t possible. It’s  terrible.” |
