# Supplementary material for: How can post-COVID care be improved using patient experiences with received care and perceived health? A qualitative study of focus groups with 30 patients having post-COVID in the Netherlands
Source: BMJ Open. 2025 Sep 21;15(9):e090771. doi: 10.1136/bmjopen-2024-090771 (PMC12458768; doi:10.1136/bmjopen-2024-090771)
Supplement: Supplementary file 4 [file bmjopen-2024-090771.reviewer_comments.pdf]

PEER REVIEW HISTORY

BMJ Open publishes all reviews undertaken for accepted manuscripts. Reviewers are asked to complete a checklist review form and are provided with free text boxes to elaborate on their assessment. These free text comments are reproduced below.

ARTICLE DETAILS

Title (Provisional)

How can post-COVID care be improved using patient experiences with received care and perceived health? A qualitative study of focus groups with 30 post-COVID patients in the Netherlands

Authors

Gruiskens, Jeroen; Gidding-Slok, Annerika HM; van Meulenbroek, Thijs; Burgers, Jako S.; Huijnen, Ivan; Muris, Jean WM; Verbunt, Jeanine; van Schayck, Onno CP

VERSION 1 - REVIEW

**Reviewer**	**1**
**Name**	**Sunkersing, David**
**Affiliation**	**University College London, Institute of Health Informatics**
**Date**	**30-Aug-2024**
**COI**	**N/A**

Dear Authors,

I have reviewed your manuscript and am pleased to report that it is well-written and addresses an important and timely topic. Your work provides valuable insights and contributes meaningfully to the current body of knowledge in this area.

Comments:

• Under 'Data Collection', it is mentioned that 'Two participants had to drop out during the interviews because they felt emotionally overwhelmed and overloaded.'. Was any support given to these participants and/or were they directed/provided with additional resources? Can you also add whether any of their data is used in the results, or whether this has been removed entirely.

• Were participants paid for their time, or involvement in the study? What implications does this have? Something to discuss, perhaps in limitations?

• Is the gender split of participants something that should be explicitly discussed in the limitations? How does this compare with current literature (either in Holland/globally)?

• It would be interesting to see a more detailed breakdown of the age (years) of participants.

• In II.2 Satisfaction with current care provision – I am not sure the quote is representative of what is being said in the paragraph. I suggest another one better related to the paragraph is used.

• In III.2. Knowledge management and organization of care – it is mentioned that 'Moreover, care should also be reimbursed properly'. Has the correct word (reimbursed) been used here, or should the sentence be something like 'Moreover, care should also be charged fairly'. If 'reimbursed' is the correct word, who is doing the reimbursing?

• In III.2. Knowledge management and organization of care – the quote, I think a square bracket is needed within the quote used to expand upon what 'nobody talks to each other' is referring to. Does this refer to the healthcare professionals? It is currently unclear.

• I was surprised to see that digital post-COVID care did not feature as something mentioned or highlighted by the patients. Was this not brought up? In our study (https://bmjopen.bmj.com/content/14/5/e080967) we noted that digital/hybrid care approaches were being used in some aspect as a constituent of post-COVID care. If this is not as common in Holland, perhaps it would be useful to mention in the paper that while these approaches are not as common in Holland (or among your sample), they are being actively used as post-COVID care approaches in other countries, such as in England.

• Supplementary reporting needs to be completed (e.g. funding details for the study were not given, competing interests not given and patient and public involvement not given).

• Though the authors have stated that the study was approved by the local Ethical Review Board, the paper would benefit from having a full 'Ethics Approval' statement at the end of the paper.

Overall, the manuscript is a strong contribution to the literature, and with some minor revisions, it will be even more impactful. I encourage you to address the points mentioned

above to improve the manuscript further. Thank you for the opportunity to review this work, and I look forward to seeing the final version.

VERSION 1 - AUTHOR RESPONSE

A. Under 'Data Collection', it is mentioned that 'Two participants had to drop out during the interviews because they felt emotionally overwhelmed and overloaded.'. Was any support given to these participants and/or were they directed/provided with additional resources? Can you also add whether any of their data is used in the results, or whether this has been removed entirely.

Reply: Before each focus group, we explained we understood and appreciated the effort and time each participant was willing to invest in the focus group. We explained we understood that due to their condition, we wanted to act in their best interest and if a participant felt they had to recuse themselves from the further discussion due to their health issues, that would be ok. The focus group's participants were very empathic and cognizant of their fellow participants and we comforted them it was fine to leave. We asked if they needed further support, and they could reach out to us if they required anything else. The two participants in question explained they needed rest and had their support structure in place, so no additional resources were provided. We invited them to feel free to send us a mail if they wished to contribute anything outside of the focus group and wished them well. The data was used in the results as indicated the pervasive nature of the Post-COVID Syndrome and its psychosocial burden. We added this to the section Data collection.

> The data from these participants was included in the analysis to help understand the pervasive nature of the post-COVID condition and its psychosocial burden.

B. Were participants paid for their time, or involvement in the study? What implications does this have? Something to discuss, perhaps in limitations?

Reply: The patients received no payment for their time or involvement in the study. The participants were eager to contribute to future improved post-COVID care, and both their motivation to improve care as well as frustration and dissatisfaction with current care seemed to be primary reasons for participation. We sampled the participant population according to patient complexity capturing a variety of perceived patient burdens in our sample. However, all patients were recruited from a national post-COVID patient platform, so one could expect patients to be engaged which might be a sub-sample of the total population. We added to the limitations of our study.

> Although we were satisfied with the sample recruited, we sampled patients from an active post-COVID patient platform which might lead to some bias, as patients involved in such a platform usually are more engaged.

C. Is the gender split of participants something that should be explicitly discussed in the limitations? How does this compare with current literature (either in Holland/globally)?

Reply: To our knowledge female gender is associated with post-COVID syndrome. We included several references in the manuscript of research associating female gender with persistent symptoms after a COVID-19 infection. We also added our predominantly female sample population as a limitation as gender might influence symptomatology, recovery, and care needs. It would be interesting to list this as a research opportunity.

> Although persistent post-COVID symptoms are more common in female patients (80, 81), and our sample predominantly was female, valuable insights on gender differences in care experiences and outcomes might be missing from our research and could be a topic for future research.

D. It would be interesting to see a more detailed breakdown of the age (years) of participants.

Reply: We received word from the editor that a more detailed breakdown of the age of the participants would be a breach of anonymity policy of BMJ Open.

E. In II.2 Satisfaction with current care provision – I am not sure the quote is representative of what is being said in the paragraph. I suggest another one better related to the paragraph is used.

Reply: Replaced the quote from another of the comprehensive data table. Please note that the first-order categories supported by quotes in the data table together contribute to the second-order theme we named Satisfaction with current care provision.

> Participant 4, female: *"I won't do physiotherapy again, it really destroyed me during the initial phases of my recovery. ... My physiotherapist did a sort of e-learning course for post-COVID. I worked out twice a week for half a year with her guidance, and that went well, but after therapy, I was bedridden for the whole week. ... After I stopped the physiotherapy, my health increased significantly."*

F. In III.2. Knowledge management and organization of care – it is mentioned that 'Moreover, care should also be reimbursed properly'. Has the correct word (reimbursed) been used here, or should the sentence be something like 'Moreover, care should also be charged fairly'. If 'reimbursed' is the correct word, who is doing the reimbursing?

Reply: The term reimbursement is used here as in The Netherlands insurance companies reimburse the healthcare professional using various insurance schemes. Such schemes did not fully cover the care desired or needed by patients and often were restricted by a cap on how many treatment sessions were being reimbursed. Patients were confronted by not being covered by insurance schemes or limited to an insufficient number of treatments given to them to aid them in a long recovery process. Furthermore, activities from healthcare professionals such as networking and dissemination of knowledge amongst professionals are currently not reimbursed by the insurance companies. We tried to clarify this in the paragraph.

> as in some cases, participants mentioned that therapy was inaccessible due to financial constraints when their insurance companies refused further reimbursement of healthcare professionals consulting due to their policy.

G. In III.2. Knowledge management and organization of care – the quote, I think a square bracket is needed within the quote used to expand upon what 'nobody talks to each other' is referring to. Does this refer to the healthcare professionals? It is currently unclear.

Reply: Clarified this in the manuscript.

> *[Talking about communication between different healthcare professionals].*

H. I was surprised to see that digital post-COVID care did not feature as something mentioned or highlighted by the patients. Was this not brought up? In our study (https://bmjopen.bmj.com/content/14/5/e080967) we noted that digital/hybrid care approaches were being used in some aspect as a constituent of post-COVID care. If this is not as common in Holland, perhaps it would be useful to mention in the paper that while these approaches are not as common in Holland (or among your sample), they are being actively used as post-COVID care approaches in other countries, such as in England.

Reply: We are aware that digital post-COVID care is becoming more common currently. At the moment of data collection, we spoke with patients during the time post-COVID care was nascent and still very fragmented. For example, using the results of this study we developed a post-COVID digital assistance tool to help measure patient burden, facilitate person-centered care and support shared decision making. Thank you for your reference. We included it in the discussion section, stating that such tools are being more actively used. At the time of data collection, however, none of the patients were using digital post-COVID care.

> Currently, the use of digital post-COVID care is actively used in many health systems (79).

I. Supplementary reporting needs to be completed (e.g. funding details for the study were not given, competing interests not given and patient and public involvement not given).

Reply: We were under the impression these details should be provided on the Title page. Our apologies, we added them at the end of the document.

Funding statement: This work was supported by ZonMw grant number 50-56300-98-6061
Competing interests: This research reports no competing interests

J. Though the authors have stated that the study was approved by the local Ethical Review Board, the paper would benefit from having a full 'Ethics Approval' statement at the end of the paper.

Reply: We added an Ethics Approval statement as requested.

Ethics Approval Statement:

1. Ethical approval for this study was obtained from the Maastricht University Medical Centre+ Ethical Review Board (METC- 2022-3439)
2. Written informed consent was obtained for anonymized patient information to be used and published in this article
